# Qualitative study on influencing factors of acceptance of advance care planning in patients with end-stage heart failure based on COM-B theory

**DOI:** 10.3389/fpubh.2026.1820593

**Published:** 2026-07-27

**Authors:** Na Liu, Lina Chen, Xinrui Mao, Shuqin Xia, Qi Wang, Dandan Hu, Weiying Zhang

**Affiliations:** 1 School of Medicine, Tongji University, Shanghai, China; 2 Department of Emergency, Shanghai East Hospital, Shanghai, China; 3 School of Nursing, Hubei University of Medicine, Shiyan, Hubei, China; 4 Department of Nursing, Shanghai East Hospital, School of Medicine, Tongji University, Shanghai, China

**Keywords:** advance care planning, COM-B, heart failure, patient acceptance of health care, qualitative research

## Abstract

**Objective:**

Patients with end-stage heart failure (ESHF) face high mortality and complex decision-making needs, yet advance care planning (ACP) acceptance is insufficient in clinical practice. This study aimed to explore factors influencing ACP acceptance among ESHF patients based on the COM-B model, focusing on knowledge deficits, familism, filial piety, and death taboo, to provide evidence for developing culturally adapted ACP interventions in China.

**Methods:**

A descriptive phenomenological qualitative design was used. Twenty-two ESHF patients were recruited via purposive sampling from a tertiary hospital. Semi-structured in-depth interviews were guided by the COM-B framework. Data were analyzed using Colaizzi’s seven-step phenomenological method for coding and theme extraction.

**Results:**

Three core themes and nine sub-themes were identified: (1) Capability: severe lack of disease and ACP knowledge, weak information comprehension and decision-making capacity, and limited information access; (2) Opportunity: family-centered decision-making restricting patient autonomy, filial piety leading to communication avoidance, inadequate medical communication, and cultural taboos causing moral pressure; (3) Motivation: fear triggered by death taboo, weakened autonomous motivation due to filial responsibility, and low perceived benefits of ACP.

**Conclusion:**

ACP acceptance among ESHF patients is shaped by cognitive capability, family culture, clinical support, and internal motivation. Insufficient knowledge is the fundamental barrier, and familism and filial piety act as key structural constraints. Culturally adapted interventions based on the COM-B model can effectively improve ACP acceptance and decision-making quality. The findings are transferable to ACP practice in other chronic conditions and collectivist cultural settings.

## 1 Introduction

Heart failure represents the end stage of various cardiac diseases. Patients with end-stage heart failure (ESHF) experience recurrent conditions, poor prognoses, and high mortality rates, often facing urgent medical decisions and risks of futile treatment (1–4). Advance care planning (ACP) refers to the process in which patients, while conscious, discuss their future medical preferences, treatment goals, and care wishes with healthcare providers and family members (5). It safeguards patients’ autonomy, improves the quality of end-of-life care, and reduces the waste of medical resources (3, 6–8). In recent years, ACP has been gradually promoted in the field of end-stage chronic diseases in China (9). However, the acceptance of ACP among ESHF patients remains generally low (10–14). Quantitative studies have mostly revealed correlations between influencing factors but failed to explain the deep-seated cultural and psychological mechanisms underlying decision-making.

The purpose of this study is to explore the deep-seated reasons for low ACP acceptance among ESHF patients and to provide a theoretical basis for the construction of cultural-adapted intervention programs.

Internationally, ACP has been widely applied in patients with malignant tumors, renal diseases, heart failure and other end-stage diseases, and its effectiveness in improving the quality of end-of-life care has been widely confirmed (5, 15, 16). However, most relevant studies are based on Western individual-oriented cultural backgrounds, and their conclusions cannot be directly extended to China’s family-oriented medical decision-making model.

In China, the implementation of ACP is profoundly affected by traditional culture. Familism, filial piety, and death taboo jointly shape patients’ attitudes toward ACP and form unique cultural barriers (17–19). At the same time, patients generally lack disease and ACP-related knowledge, which further increases the difficulty of decision-making (20).

In recent years, although standardized ACP intervention models have been established in fields such as cancer and nephropathy, there is still a lack of targeted qualitative research that integrates cultural characteristics in the field of heart failure. A study in 2025 pointed out that the implementation of ACP can significantly reduce the invalid resuscitation rate of patients with heart failure, but China still lacks a unified and culturally adapted implementation plan (9).

In view of the complexity of ACP acceptance behavior, the Capability-Opportunity-Motivation-Behaviour (COM-B) model is introduced in this study. The model holds that behavior is produced by the interaction of individual capability, external opportunity, and internal motivation, and can systematically explain complex health decision-making behaviors (21). Compared with the Theory of Planned Behavior and Health Belief Model, the COM-B model can more comprehensively cover the cognitive, familial, cultural and clinical factors of ACP behavior, making it more suitable for this study.

Based on the COM-B model and the background of Chinese family culture, this study aims to deeply explore the influencing factors and mechanism of ACP acceptance in patients with ESHF, focusing on the roles of insufficient knowledge, familism, filial piety culture, and death taboo. The findings are expected to provide theoretical and practical evidence for constructing culturally adapted ACP intervention strategies, and to improve the ACP acceptance rate and decision-making quality of patients with ESHF.

Specific research questions

1) What capability-related factors affect ACP acceptance in patients with end-stage heart failure?
2) What opportunity-related barriers including family culture, medical communication, and cultural taboos hinder ACP participation?
3) What motivational factors including emotional fear, filial moral pressure, and perceived benefits reduce patients’ willingness to use ACP?
4) What culturally appropriate interventions can be developed to promote ACP acceptance based on the study findings?

This study was approved by the hospital ethics committee (Approval No.: 2025YS095). All participants signed written informed consent, and the study was conducted in strict accordance with the Declaration of Helsinki.

## 2 Subjects and methods

### 2.1 Study design and participants

This study employed a descriptive phenomenological qualitative design to explore patients’ lived experiences of advance care planning (ACP) acceptance. The study was conducted under the epistemological perspective of interpretivism, which focuses on understanding individuals’ subjective experiences and perceptions. Data were analyzed using Colaizzi’s seven-step method (22). This study was reported in accordance with the COREQ (Consolidated Criteria for Reporting Qualitative Research) guideline, and the COREQ checklist is provided as supplementary material (23). A purposive sampling method was used to select patients hospitalized with ESHF in the Department of Cardiology and Coronary Care Unit (CCU) of a tertiary hospital from Jan 2026 to Feb 2026. Inclusion criteria: (1) Met the diagnostic criteria for ESHF, with NYHA class III–IV cardiac function (24); (2) Aged ≥18 years; (3) Clear consciousness and normal communication; (4) Aware of their condition, voluntarily participated and signed informed consent. Exclusion criteria: (1) Severe cognitive impairment or mental illness; (2) critically ill and unable to cooperate with interviews; (3) language communication barriers. The sample size was determined based on information saturation, and 22 patients were finally included, including 13 males and 9 females; aged 54–88 years; education level: primary school and below 10 cases, middle school 8 cases, junior college and above 4 cases; disease course 1–10 years.

### 2.2 Data collection method

Data were collected through face-to-face, semi-structured in-depth interviews conducted in quiet and private rooms. Each interview was conducted by two nursing postgraduate students who had received systematic training in qualitative research and cultural sensitivity. A neutral attitude was maintained to avoid leading questions. Participants were informed of the research purpose, confidentiality, and audio recording. All interviews were audio-recorded, and non-verbal information was documented. Each interview lasted 25–45 min, and verbatim transcription was finished within 24 h.

### 2.3 Interview guide

According to the research purpose and the COM-B model, a semi-structured interview outline was compiled, and relevant literature on advance care planning (ACP) was systematically reviewed. Three nursing and palliative care experts were consulted to judge whether the interview outline comprehensively covered the research scope, whether any content deviated from the research topic, and whether the wording of each question was clear and accurate. All experts independently put forward suggestions for revision. The research team held a meeting to discuss all comments and revised the preliminary draft accordingly, including optimizing question expressions, adjusting the logical sequence, and ensuring cultural sensitivity for end-of-life topics. Then, pre-interviews were conducted with 3 participants, and further adjustments were made based on their feedback to ensure clarity, understandability, and clinical suitability. The core questions of the final interview outline are presented in Table 1.

**Table 1:** Interview guide.

Interview questions
Capability
1. Have you heard of “advance care planning” or “living will”? How do you understand its role?
2. When making decisions about future treatment or care, do you think you have enough information? What content do you find difficult to understand?
3. What do you know about the possible future changes of your disease? How does this knowledge affect whether you think advance planning is necessary?
Opportunity
4. When discussing future treatment with your family, what factors make communication easy or difficult?
5. Have medical staff ever talked to you about future medical options? In what ways do you hope they can provide more support?
Motivation
6. When talking about future treatment or life care, what emotions or concerns do you have? Do these emotions encourage you to participate in ACP or make you hesitate?
7. What benefits or worries do you think advance care planning has? How do these views affect your willingness to participate in ACP?
Suggestions for improvement
8. How do you think hospitals or society can improve to better support patients in completing such planning?

### 2.4 Research team

The research team was composed of experienced nursing researchers and postgraduate nursing students. The first and second authors were mainly responsible for study design, participant interviews, and data analysis. During each interview, one researcher conducted the face-to-face conversation, while the other assisted with observation and field notes.

The two interviewers were full-time researchers with no clinical management duties and had no hierarchical or therapeutic relationship with the participants. All team members participated in discussions of coding discrepancies. When consensus could not be reached, the corresponding author, a senior professional in nursing and palliative care, made the final decision to ensure the scientific rigor and accuracy of data analysis.

All researchers had received systematic training in qualitative research methods and cultural sensitivity, with rich experience in end-of-life care research, ensuring the rigor and credibility of the study.

### 2.5 Data analysis method

Data were analyzed using Colaizzi’s seven-step phenomenological method (22),which included: familiarizing with transcripts, extracting meaningful statements, coding, clustering themes, generating detailed descriptions, constructing the fundamental structure, and verifying the structure. Researchers repeatedly read the transcripts to become familiar with the data. To ensure rigor, two trained researchers independently conducted initial coding and theme extraction. Discrepancies during coding were resolved through group discussion until consensus was reached. An audit trail and standardized coding manual were established to ensure analytical transparency. Data saturation was achieved after 18 interviews, with no new themes emerging; 4 additional interviews were conducted to confirm saturation, and 22 participants were finally included. Member checking was performed with 6 participants to verify that themes reflected their real experiences. All participants confirmed the accuracy and authenticity of the findings. Reflexivity was maintained throughout the research process to reduce potential bias in interpreting cultural factors.

### 2.6 Rigor

This study was conducted in strict accordance with the COREQ guidelines for qualitative research (23). To guarantee the scientific rigor and reliability of the entire research process, the research team established a clear division of labor and completed specialized training prior to participant recruitment. Before formal interviews, appropriate arrangements were made regarding interview time and venue to establish rapport and gain the trust of participants. During data collection, researchers maintained a neutral position throughout the process, avoiding any suggestive or leading language. To enhance research credibility, reflective practice was integrated into the entire research workflow. Immediately following each interview, two researchers independently documented field notes, with a specific focus on identifying and recording potential biases that may have arisen during the interview process. These potential biases were openly reported and collectively analyzed during regular team meetings, and corresponding corrective strategies were promptly implemented to mitigate their impact. Furthermore, to strengthen the trustworthiness of the findings, a triangulation strategy was employed. This involved cross-verification through independent researcher analysis, participant verification (member checking), and expert peer review. This multi-faceted approach effectively minimized the influence of various biases on the final research outcomes.

### 2.7 Ethical considerations

This study was approved by the Ethics Committee of Shanghai East Hospital (No. 2025YS095). Throughout the research process, all participants were fully informed of the research purpose, interview content, data usage, and privacy protection measures, and signed a written informed consent form in person. Participants were clearly informed that they had the right to withdraw from the study unconditionally and voluntarily at any time without giving any reason. After withdrawal, all their related data and recordings would be immediately deleted and would not affect their clinical treatment. All audio recordings and transcripts were used only for research and data analysis, and would not be used for any other purposes without the permission of the participants. All data were anonymized to protect participants’ privacy and confidentiality.

## 3 Results

Guided by the COM-B theoretical framework, data from semi-structured interviews with 22 end-stage heart failure patients were coded and analyzed via Colaizzi’s seven-step phenomenological analysis method. Three interwoven core themes were extracted, including capability, opportunity and motivation, alongside mutually restrictive subthemes that jointly explained patients’ low willingness to engage in advance care planning (ACP). These three dimensions did not function independently; instead, they formed a reinforcing vicious cycle, where deficits in one dimension aggravated barriers in the other two and collectively suppressed patients’ ACP acceptance.

### 3.1 Capability: deficient disease and ACP literacy constitutes the foundational barrier that restricts patients’ decision-making capacity

Insufficient cognitive capability served as the primary prerequisite barrier to ACP participation, and its restrictive effect permeated patients’ perceptions and subsequent behavioral intentions linked to opportunity and motivation. Most interviewees lacked systematic understanding of the progressive trajectory, long-term complications and prognostic risks of end-stage heart failure. Without standardized, accessible health education, patients only focused on immediate symptom relief and held widespread misconceptions that equated ACP with abandoning treatment or even euthanasia, as reflected by P5: “*I only know heart failure is serious, but doctors never told me what will happen later. I thought advance planning means giving up treatment, so I dare not even think about it, let alone sign relevant documents.”*

Limited health comprehension further weakened patients’ autonomous decision-making capacity. Owing to advanced age, low educational attainment and heavy reliance on specialized medical jargon, participants struggled to grasp the meaning of rescue interventions and prognostic assessments. Many reported being unable to independently judge treatment trade-offs and passively yielded to their family’s choices (P11: *“I cannot understand or remember those professional words at all, so I have no way to make my own decisions and can only follow my family and doctors’ advice”*). In addition, patients’ access to targeted ACP information was extremely fragmented: they only obtained scattered explanations during occasional ward rounds, with no structured, easy-to-read educational materials available in the ward environment. As P16 described, “*No staff has systematically explained these things to me, and I have nowhere to turn for answers when I have questions.*” This single-channel, disorganized information supply deepened cognitive biases and left patients incapable of forming complete, objective judgments of ACP value.

### 3.2 Opportunity: familial collectivism and cultural taboos create structural communication barriers that cut off ACP discussion channels

Against China’s family-centered medical decision-making culture, multiple intertwined opportunity barriers blocked patients’ chances to communicate end-of-life care preferences, and such external constraints amplified the negative impacts of insufficient cognitive capability while dampening internal motivation. The most prominent structural constraint stemmed from familism: all participants viewed medical choices as a family affair and believed individual medical preferences must be subordinate to collective family opinions, leaving little room for them to voice independent wishes. P2 stated, “*I cannot make decisions alone; everything has to follow my children’s thoughts. I do not want to embarrass them, so I will not bring up such topics if my family disagrees.*”

Rooted in traditional filial piety norms, family members actively avoided conversations about prognosis and end-of-life planning out of protective psychology, further closing off ACP communication opportunities. P9 shared that *her children deliberately evaded related topics to prevent her from feeling sad, which in turn made her hesitant to initiate discussions voluntarily*. Beyond familial barriers, inadequate clinical communication further narrowed available opportunities to talk through ACP: medical staff only brought up end-of-life treatment options when patients experienced acute exacerbations. At such stressful moments, patients felt flustered and emotionally resistant to relevant conversations (P18: “*They only mention these things when I am severely ill. I feel too upset to listen or talk through it calmly*”). Layered on top of family and clinical communication limitations, the cultural death taboo imposed additional moral pressure on patients. Many regarded proactive discussion of future care as inauspicious, and internalized social and familial disapproval made them reluctant to explore ACP-related topics openly (P7: “*Talking about end-of-life treatment in advance feels unlucky, and my whole family is opposed to it, which also makes me uncomfortable to think about*”). Collectively, family culture, untimely clinical communication and cultural taboos formed overlapping external obstacles that deprived patients of feasible spaces to express their medical preferences.

### 3.3 Motivation: death-related fear and moral burden suppress internal willingness to participate in ACP

Restricted cognitive capability and blocked external communication opportunities jointly undermined patients’ intrinsic motivation to pursue ACP, while death avoidance psychology and filial moral burden acted as direct inhibiting factors, creating a self-reinforcing negative loop of low acceptance. Driven by the widespread death taboo, nearly all interviewees reported intense fear and evasive emotions when discussing end-of-life interventions such as invasive intubation and emergency resuscitation. Framing ACP as a direct confrontation with mortality triggered strong psychological discomfort, as P19 noted: “*Just thinking about intubation and rescue scares me; planning ahead feels like sentencing myself to death, which makes me extremely uncomfortable*.”

Beyond death anxiety, the moral obligation of filial responsibility further weakened patients’ autonomous motivational drive. Many participants voluntarily surrendered their right to make medical decisions out of concern that their independent end-of-life choices would lead their children to be judged as unfilial by relatives and acquaintances. P14 explained, “*I cannot make these decisions on my own, otherwise others will say my children are not filial, so I choose not to bring up such matters for their sake.*” Compounding emotional and moral suppression, most patients failed to perceive tangible personal benefits from ACP. They held the view that future rescue plans would be fully determined by their real-time physical condition, rendering advance planning meaningless (P3: “T*here is no point talking about this now; doctors will adjust treatment according to my illness then, so there is no need to bother planning ahead*”). Low perceived benefit further eliminated patients’ proactive willingness to seek out ACP communication, and this lack of motivation in turn reduced their initiative to acquire relevant disease and care knowledge, perpetuating the cycle of insufficient capability and limited opportunity identified in the previous two themes.

## 4 Discussion

This qualitative study adopted the COM-B behavior change framework proposed in prior theoretical research to systematically unpack intertwined barriers shaping patients’ advance care planning (ACP) acceptance among Chinese end-stage heart failure patients within familistic and filial cultural contexts. Rather than treating capability, opportunity and motivation as separate isolated predictors, interview data indicated that the three dimensions interact reciprocally and form a reinforcing vicious cycle that suppresses patients’ willingness to participate in end-of-life medical communication, which matches the core explanatory logic of the COM-B model and the findings of existing domestic qualitative studies targeting chronically ill populations (25).

Insufficient disease and ACP literacy constitutes the fundamental cognitive barrier that restricts patients’ independent medical decision-making capacity, and such cognitive deficits will generate cascading negative impacts on external communication opportunities and internal participation motivation simultaneously. Most interviewees held biased perceptions that equated ACP with giving up treatment or euthanasia, a consistent phenomenon reported in previous qualitative studies of older patients with chronic diseases and tumour patients (19, 26). Advanced age and limited educational background further made it difficult for patients to comprehend professional prognostic and resuscitation knowledge, leading them to defer all medical choices to their families. Meanwhile, patients could only acquire fragmented ACP information from ward rounds without standardized popular science materials to correct cognitive biases. Such insufficient cognitive capacity cannot be regarded as an independent obstacle; even if hospitals provide sufficient communication opportunities, patients without basic understanding of ACP will still show obvious avoidance and lack active information-seeking willingness, which conforms to the precondition logic of capability in COM-B theory (27).

Restricted external opportunity constructed by familism, filial avoidance and death taboos forms structural environmental constraints, which further amplify the adverse effects of cognitive deficiency and weaken patients’ internal participation motivation. Medical decision-making in China takes family as the core unit, and all participants regarded treatment choices as collective family affairs that override individual medical preferences, leaving little room for patients to express independent wishes (28). Driven by filial piety, family members deliberately avoid prognosis-related conversations out of protective psychology, further blocking ACP communication channels, which has also been verified in domestic palliative care research (19). In clinical practice, medical staff only mention end-of-life schemes during acute exacerbations when patients are emotionally agitated and unable to engage in calm discussion. Together with the cultural taboo of talking about death in advance, multiple overlapping barriers cut off effective communication space for patients. These environmental constraints interact bidirectionally with cognitive deficits: patients with inadequate ACP cognition are more likely to accept their families’ avoidant attitudes, a kind of interactive restriction rarely observed in Western individualistic medical contexts (5).

Suppressed internal motivation is the direct psychological result of insufficient capability and blocked communication channels. Death avoidance psychology and filial moral burden jointly inhibit patients’ willingness to participate in ACP, and this negative emotion will in turn reduce patients’ initiative to supplement disease knowledge, thus consolidating the vicious cycle. Almost all respondents reported strong discomfort when discussing intubation and rescue measures, and they equated ACP with facing death directly (26). Many patients voluntarily gave up decision-making power to prevent their children from being judged unfilial by relatives. In addition, most patients failed to perceive tangible benefits of advance planning and held the view that real-time condition assessment can replace pre-planning, which further eliminated active participation intention. The reciprocal restrictive relationship among capability, opportunity and motivation fully verifies the applicability of COM-B model to Chinese end-of-life decision scenarios, and the filial cultural constraints reflected in the findings are significantly weaker in Western research samples (29).

**Figure 1:**
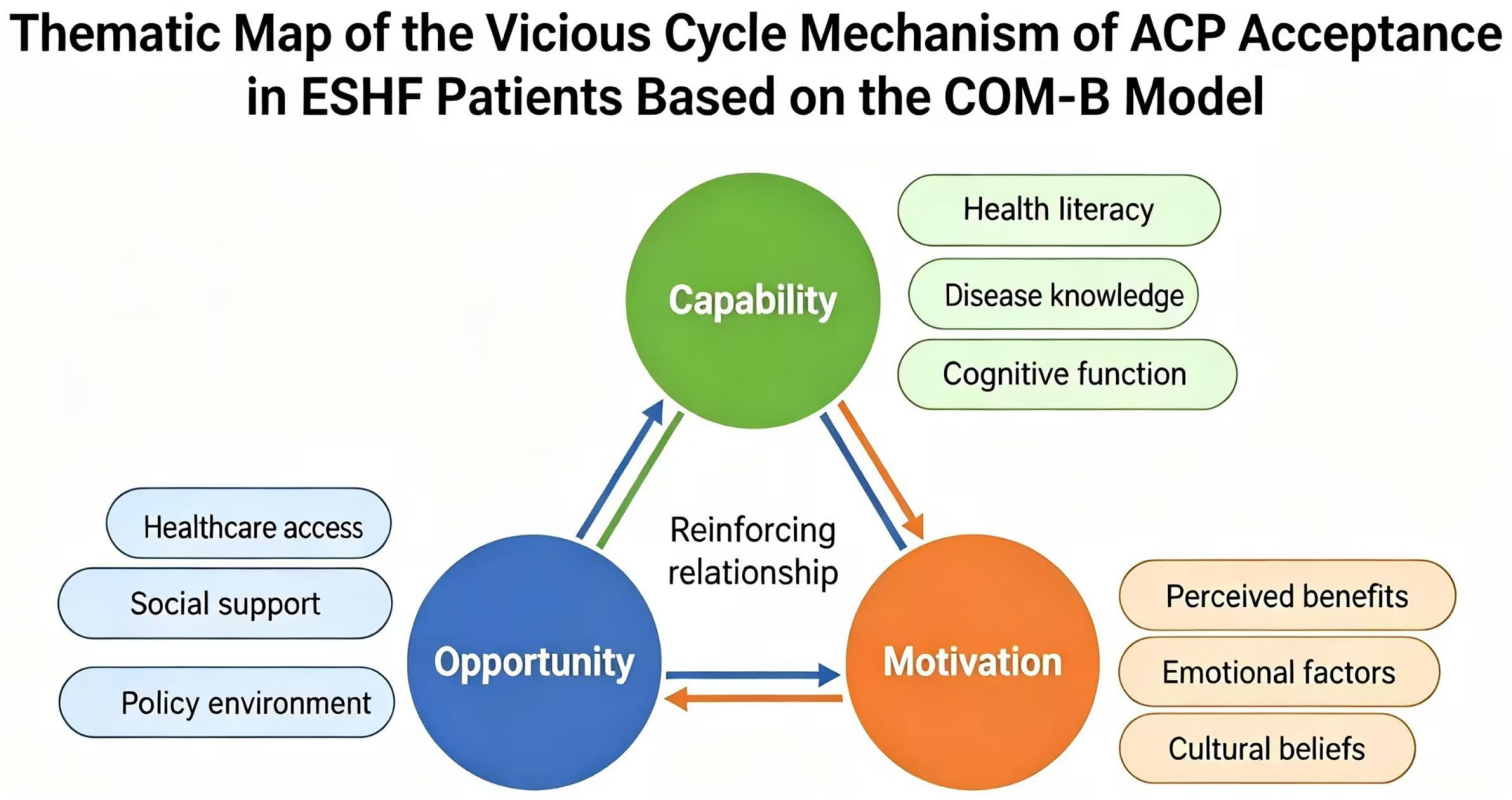
Simplified thematic map of the reinforcing cycle mechanism of ACP acceptance. Actual interactions are moderated by individual factors (age, education), clinical conditions, family function, and institutional support. This figure illustrates the interactive vicious cycle among capability, opportunity, and motivation. Arrow indicate the reinforcing relationships between the three core themes, with sub-themes, with sub-themes nested under each core theme to show the formation mechanism of low ACP acceptance.

Based on the three-dimensional cyclic interaction mechanism summarised in this study, culturally localised ACP intervention strategies can be put forward, which cannot directly copy Western outpatient individual consultation modes (30). Hospitals should take correcting cognitive bias as the primary measure and carry out simplified, targeted health education for patients. Meanwhile, medical staff need to adjust ACP communication timing to stable disease periods and adopt family joint interview modes to break intergenerational avoidance of end-of-life topics. During communication, ACP should be interpreted as a manifestation of rational filial respect rather than abandoning treatment to relieve patients’ moral pressure. Only simultaneous interventions targeting the three dimensions can fundamentally break the vicious cycle limiting ACP acceptance (25) (Figure 1).

### 4.1 Limitations

This study had several limitations. First, a single-center design and purposive sampling were adopted, and the sample size was relatively small, which might limit the diversity and representativeness. Second, only patients’ perspectives were included, while the views of family members and medical staff were not obtained, which may restrict the interpretation of the opportunity component in the COM-B model. Third, all participants were from urban eastern China; thus, the findings might not be fully generalizable to rural areas or other economic and cultural regions. Fourth, although data saturation was achieved, the exploratory qualitative design meant that the conclusions supported theoretical transferability rather than empirical generalization. Finally, the application of the COM-B model provided a clear framework, but it might also constrain the emergence of potential themes to a certain extent. These limitations provided directions for future multi-center, mixed-method, and multi-stakeholder studies.

### 4.2 Transferability and suggestions for future research

Although this study was conducted in patients with end-stage heart failure, the theoretical framework and cultural mechanism had certain transferability. The influencing factors such as knowledge deficit, family decision-making, filial piety, and death taboo also existed in other end-stage chronic diseases (e.g., advanced cancer, end-stage renal disease) and other collectivist cultural regions. Future research could carry out multi-center studies, include family members and medical staff, and conduct longitudinal intervention studies based on the COM-B model to verify the effectiveness of culturally adapted ACP programs.

## 5 Conclusion

This qualitative study based on the COM-B model explored ACP acceptance among patients with end-stage heart failure in urban eastern China. Within this specific regional and cultural context, ACP acceptance is jointly determined by capability (knowledge deficit), opportunity (family and cultural constraints), and motivation (emotional and moral inhibition). Insufficient knowledge serves as the fundamental barrier, while familism and filial piety constitute key structural constraints.

These findings are contextually rooted in Chinese collectivist culture and should not be broadly generalized to other cultural settings without adaptation. The conclusions support the development of culturally congruent ACP interventions rather than standardized Western models. With improved education, family-involved communication, and culturally sensitive support, ACP acceptance can be enhanced to protect patient autonomy and improve end-of-life care quality for patients with end-stage heart failure.

## Data Availability

The original contributions presented in the study are included in the article, further inquiries can be directed to the corresponding author.
